# SEOM clinical guidelines in Hereditary Breast and ovarian cancer

**DOI:** 10.1007/s12094-015-1435-3

**Published:** 2015-12-15

**Authors:** G. Llort, I. Chirivella, R. Morales, R. Serrano, A. Beatriz Sanchez, A. Teulé, E. Lastra, J. Brunet, J. Balmaña, B. Graña, On behalf of the SEOM Hereditary Cancer Working Group

**Affiliations:** Unidad de Consejo Genético, Servicio de Oncología Médica, Corporació Sanitària Parc Taulí/Consorci Sanitari de Terrassa, Parc Taulí s/n, 08208 Sabadell, Barcelona Spain; Oncología Médica, Hospital Clínico Universitario de Valencia, Valencia, Spain; Servicio de Oncología Médica, Hospital La Mancha Centro, Ciudad Real, Spain; Unidad de Consejo Genético, Servicio de Oncología Médica, Hospital Universitario Reina Sofía (Córdoba), Córdoba, Spain; Unidad de Consejo Genético en Cáncer, Servicio de Oncología Médica, Hospital General Universitario de Elche, Alicante, Spain; Unidad de Consejo Genético, Programa de Cáncer Hereditario, Institut Català d’Oncologia-IDIBELL, L’Hospitalet del Llobregat, Barcelona, Spain; Unidad de Consejo Genético, Sección Oncología Médica, Hospital Universitario de Burgos, Burgos, Spain; Unidad de Consejo Genético, Programa de Cáncer Hereditario, Institut Català d’Oncologia-IDIBGI, Girona, Dpto Ciencias Médicas, Facultad de Medicina, Universidad de Girona, Girona, Spain; Programa Cáncer Familiar, Servicio Oncología Médica, Hospital Vall d’Hebron, Barcelona, Spain; Unidad de Alto riesgo en Cáncer, Servicio de Oncologia Médica, Complejo Hospitalario Universitario A Coruña (CHUAC), La Coruña, Spain

**Keywords:** Hereditary breast and ovarian cancer, BRCA1 and BRCA2 genes, Prevention, SEOM

## Abstract

Approximately, 7 % of all breast cancers (BC) and 11–15 % of ovarian cancers (OC) are associated with inherited predisposition, mainly related to germline mutations in high penetrance BRCA1/2 genes. Clinical criteria for genetic testing are based on personal and family history to estimate a minimum 10 % detection rate. Selection criteria are evolving according to new advances in this field and the clinical utility of genetic testing. Multiplex panel testing carries its own challenges and we recommend inclusion of genes with clinical utility. We recommend screening with annual mammography from age 30 and breast MRI from age 25 for BRCA1 and BRCA2 mutation carriers. Bilateral salpingo-oophorectomy should be offered to women with a BRCA1 or BRCA2 mutation, between 35 and 40 years and after completion of childbearing, or individualise based on the earliest age of ovarian cancer diagnosed in the family. Bilateral risk-reducing mastectomy is an option for healthy BRCA1 and BRCA2 mutation carriers, as well as contralateral mastectomy for young patients with a prior BC diagnosis. BRCA genetic testing in patients with BC and OC may influence their locoregional and systemic treatment.

## Hereditary breast and ovarian cancer syndrome (HBOC): introduction

Approximately, 7 % of all breast cancers (BC) and 11–15 % of ovarian cancers (OC) are associated with inherited predisposition, mainly related to germline mutations in high penetrance BRCA1/2 genes. A meta-analysis reports mean cumulative BC risk at age 70 years of 57 % (95 % CI 47–66) for *BRCA1* and 49 % (95 % CI 40–57) for *BRCA2* mutation carriers; and OC risk of 40 % (95 % CI 35–46) for BRCA1 and 18 % (95 % CI 13–23) for BRCA2 mutation carriers [1, 2].

Advances in sequencing technologies make massive parallel sequencing more feasible and afford testing for other hereditary predisposition genes assigned to high BC risk (*TP53*, *PALB2*, *PTEN*), moderate BC risk (*CHEK2*, *ATM*, *NF1*, *NBN*), elevated, but imprecise BC risk (*CDH1*, *STK11*) and OC risk (MMR genes, *RAD51D*, *BRIP1*) [3].

HBOC families associated to *BRCA1* or *BRCA2* germline mutations present an autosomal dominant hereditary pattern, with early ages of cancer onset, bilaterality and male breast cancer. *BRCA1*-associated BC usually have a higher histological grade and a triple-negative basal phenotype. OC in *BRCA1* and *BRCA2* mutation carriers are high-grade serous adenocarcinoma with intraepithelial infiltrates, lymphocytic atypia and abundant mitoses.

## Criteria for BRCA genetic testing

We recommend genetic counselling pre- and post-germline genetic testing. Genetic counselling is a process that guarantees a discussion about the benefits and limitations of genetic testing, provides risk estimates of developing cancer, recommendations for early detection and preventive measures, information about reproductive options and support for psychological well-being.

Clinical criteria for genetic testing are based on personal and family history to estimate a minimum 10 % detection rate [4, 5]. Hence, selection criteria are evolving according to new advances in this field and the clinical utility of genetic testing (Table 1). Multiplex panel testing carries its own challenges and we recommend inclusion of genes with clinical utility. If multiplex testing is considered for HBOC, we recommend including *TP53*, *PALB2*, *BRIP1*, *RAD51C and RAD51D* [6]. Other genes like *CDH1* and *PTEN* might be offered based on familial phenotype (bilateral lobular BC < 50, Cowden-like features) or when specific criteria for the hereditary cancer syndrome are present.

**Table 1:** Selection criteria for *BRCA* genetic testing

Regardless of family history:
Women with synchronous or metachronous BC and OC
BC ≤35 years (or BC ≤40 years in case of uninformative family^a^)
Bilateral BC (the first diagnosed ≤40 years)
Triple-negative BC ≤50 years
High-grade epithelial non-mucinous OC (or fallopian tube or primary peritoneal cancer)
2 or more first degree relatives^b^ with any combination of the following high-risk features:
Bilateral BC + another BC <50 years
Male breast cancer
BC + OC
Two cases of BC diagnosed before age 50 years
3 or more direct relatives^b^ with BC and/or OC:
≥3 BC ± OC

*BC* breast cancer, *OC* ovarian cancer

^a^Less than 2 women who have lived until age 45 or older in each side of the family

^b^In the same side of the family

## Surveillance and strategies for early detection of cancer in mutation carriers

Early detection of breast cancer aims to reduce morbidity and mortality. An individual patient data meta-analysis of high-quality observational studies shows that the use of breast MRI as an adjunct to mammography significantly increases the sensitivity of screening in women with *BRCA1 and BRCA2* mutations as compared with mammography alone (93.4 vs 39.6 %; *p* < 0.001), whereas specificity is significantly reduced (80.3 vs 93.6 %; *p* = 0.0016) [7]. Although a survival benefit from breast MRI in these women has not robustly been proven, a clear trend towards improved metastasis-free survival has been reported [8]. Therefore, we recommend annual mammography and breast MRI screening for *BRCA1* and *BRCA2* mutation carriers (IIA).

The appropriateness of imaging scheduling is still controversial. The pooled-data analysis includes women between 30 and 70 years, with MRI and mammography being performed within 1–2 months from each other [7]. In addition, radiation-induced breast cancer is a potential concern in women younger than 30 years, who also present denser breast tissue which hampers a good visualisation on mammograms. So, we recommend an annual breast MRI from age 25, with a synchronous annual mammography added after age 30 until age 70 (Table 2).

**Table 2:** Screening recommendations in BRCA mutation carriers

	Age	Evidence and recommendation
Women		
Breast self awareness	Starting at age 18 years	**IIA**
Clinical breast exam every 6–12 months	Starting at age 25 years	**IIA**
Annual breast MRI	25–70 years	**IIA**
Annual mammogram	30–35 to 75 years	**IIA**
Transvaginal ultrasound and Ca 12.5 every 6–12 months	30 years	**IIC**
Men		
Breast self awareness	Starting at age 35 years	**IIIC**
Annual clinical breast exam	Starting at age 35 years	**IIIC**
Basal mammogram	40 years (individualised)	**IIIC**
Annual Prostate Cancer screening	Starting at age 40 years	**IIIB**
Men and women		
Pancreatic and melanoma	Consider individualised screening based on cancers in the family	**IIIC**
Colorectal cancer screening, especially in BRCA1	Starting at 40 years or younger if family history	**IIB**

There are limited data to support breast imaging in men, but we recommend considering mammography at age 40 years, especially if gynaecomastia or in BRCA2 carriers (**IIIC**).

Women with a *BRCA1* or *BRCA2* mutation who have not chosen prophylactic salpingo-oophorectomy may follow determination of Ca125 and transvaginal ultrasound since age 35 (**IIC**), but they should be informed that early detection of ovarian cancer is not guaranteed.

Screening for prostate cancer at age 40 is recommended for males with a *BRCA2* mutation, due to the increased risk and poor survival outcomes, and should be individualised for *BRCA1* male mutation carriers [9] (**IIB**).

Screening for colorectal cancer with annual FIT or colonoscopy every 5 years beginning at 40 should be encouraged especially in *BRCA1* carriers (**IIIC**).

Consider individualised screening for pancreatic cancer and melanoma, based on family history.

## Risk-reducing surgery

### Bilateral salpingo-oophorectomy

The absence of reliable methods for early detection and the poor prognosis associated with advanced ovarian cancer have supported the recommendation of bilateral risk reduction salpingo-oophorectomy (RRSO) after completion of childbearing in women with *BRCA1* or *BRCA2* mutation [10]. Preventive oophorectomy was associated with an 80 % reduction in the risk of ovarian, fallopian tube or peritoneal cancer in *BRCA1* or *BRCA2* carriers and a 77 % reduction in all-cause mortality [10, 11].

A 1–4.3 % residual risk of a primary peritoneal carcinoma has been reported in some studies [12]. A meta-analysis involving 10 studies showed an approximately 80 % reduction in the risk of ovarian or fallopian cancer following RRSO. The meta-analyses also found that RRSO reduces the breast cancer risk by approximately 50 % [12].

Bilateral salpingo-oophorectomy should be offered to women with a *BRCA1* or *BRCA2* mutation, between 35 and 40 years and after completion of childbearing, or individualised based on the earliest age of ovarian cancer diagnosed in the family (**II**,**A**).

Given that short-term HT seems to improve quality of life and does not seem to increase risk of BC in healthy *BRCA* mutation carriers after bilateral prophylactic oophorectomy [13], short-term and low-dose hormone therapy in oophorectomised *BRCA* mutation carriers without a personal history of breast cancer might be considered (**II**,**B**).

### Prophylactic mastectomy

Retrospective analyses have indicated that bilateral risk reduction mastectomy (BRRM) decreases breast cancer risk by at least 90 % in *BRCA1 and BRCA2* mutation carries [10, 14].

In healthy *BRCA1* or *BRCA2* mutation carriers, BRRM reduces BC risk when compared to surveillance, while longer follow-up is warranted to confirm survival benefits [15].

In a recent prospective analysis, contralateral RRM was found to be associated with improved overall survival in *BRCA1* or *BRCA2* mutation carriers with a prior primary breast cancer (PBC). Survival benefit was especially seen in young patients (<40 years), with grade 1/2 differentiation and/or no triple-negative phenotype, and in patients not treated with adjuvant chemotherapy [16].

Bilateral risk-reducing mastectomy is an option for healthy *BRCA1* and *BRCA2* mutation carriers, as well as contralateral mastectomy for young patients with a prior BC diagnosis (**II**,**B**).

## Chemoprevention

Several case–control studies in *BRCA* mutation carriers with breast cancer show that the use of tamoxifen protects against contralateral breast cancer [17].

Adjuvant tamoxifen reduces the risk of a second breast cancer in patients with a *BRCA* mutation and a prior BC (**II**,**A**).

In the Breast Cancer Prevention Trial, healthy women with a *BRCA2* mutation receiving tamoxifen had a reduction of breast cancer by 62 %, an effect not seen among those with a *BRCA1* mutation, but the overall number of individuals was very small. There are no other chemoprevention trials in *BRCA* mutation carriers, except for an ongoing randomised clinical trial by the French Federation of Cancer Centers investigating the preventive effect of letrozole in postmenopausal women with a *BRCA1* or *BRCA2* mutation [18]. There is no demonstrated benefit for primary chemoprevention of breast cancer in *BRCA1* or *BRCA2* mutation carriers (**I**,**A**).

In a recent meta-analysis of case–control studies in *BRCA1 and BRCA2* mutation carriers, a significant 50 % risk reduction of ovarian cancer was associated with the past use of combined oral contraceptives [19]. Nevertheless, there are conflicting results on the effect of oral contraceptive use on breast cancer risk in *BRCA* mutations carriers. A recent case–control study in *BRCA1* carriers found that oral contraceptives use before age 25 increases the risk of early onset breast cancer among *BRCA1* mutation carriers and the risk increases with duration of use [20].

Use of oral contraceptives protects against ovarian cancer (**II**,**B**), but caution should be used when considering them in mutation carriers because of the conflicting results on their effect on breast cancer risk. *BRCA1* carriers should be advised to avoid oral contraceptive use if the purpose is to prevent ovarian cancer before the age of 25 (**IIB**).

## Treatment strategies in *BRCA* carriers

Breast conservative surgery in patients with early stage BC is associated with high rates of second ipsilateral recurrences, but no differences in BC-specific survival compared to mastectomy. Mutation carriers have a higher risk of contralateral BC than sporadic BC patients. Therefore, *BRCA* genetic testing in patients with early stage BC may influence their locoregional treatment (**III**,**A**).

Platinum salts have shown a high pathological complete response in the neoadjuvant setting among patients with breast cancer and a *BRCA1 or BRCA2* germline mutation [21, 22]. In the metastatic setting, carboplatin has shown a statistically clinical benefit when compared to docetaxel among *BRCA* mutation carriers [23]. Randomised phase 3 trials of PARPi for patients with *BRCA*-associated BC are ongoing.

Platinum salts might be considered in the neoadjuvant setting (**I**,**C**), and in the metastatic setting among patients with BC and a *BRCA* mutation (**I**,**A**) (Table 3).

**Table 3:** Risk reduction and therapeutic strategies in BRCA mutation carriers

Adjuvant tamoxifen reduces the risk of contralateral breast cancer (**IIA**)
Benefit of tamoxifen for primary prevention is not demonstrated in BRCA mutation carriers (**IA**)
Oral contraceptives protect against ovarian cancer (**IIB**), but caution should be used when considering use of oral contraceptives in mutation carriers because the conflicting results on their effect on breast cancer risk
Bilateral Salpingo-oophorectomy should be recommended between 35 and 40 years and upon completion of child bearing (**IA**)
Bilateral prophylactic mastectomy reduces the risk of breast cancer by at least 90 % (**IIB**), and is an option for healthy BRCA1 and BRCA2 mutation carriers, as well as contralateral mastectomy for young patients with a prior breast cancer diagnosis (**IIB**)
Platinum salts might be considered in neoadjuvant setting (**IC**) and in the metastatic setting (**IA**)
PARPi are recommended as maintenance therapy in patients with relapsed platinum-sensitive high-grade serous ovarian cancer (**IA**)

Retrospective studies have shown an improved prognosis, higher response rates and longer treatment-free intervals between relapses in patients with a *BRCA1/2*-mutated ovarian cancer treated with platinum-containing regimens compared with sporadic ovarian cancer patients [24]. These tumours also show high sensitivity to anthracyclines [25]. Poly(ADP-ribose) polymerase (PARP) inhibitors lead to synthetic lethality in HR-deficient cells and they are active drugs in patients with *BRCA1 or BRCA2*-mutated ovarian cancer. Olaparib is the first EMA-approved PARPi as a maintenance therapy in patients with relapsed platinum-sensitive high-grade serous ovarian cancer [26].

Alkylating and DNA-damaging agents are recommended for patients with ovarian cancer (**I**,**A**). PARPi are recommended as maintenance therapy in patients with relapsed platinum-sensitive high-grade serous ovarian cancer (**I**,**A**).

## Management of women without identified BRCA mutations (BRCAX)

Women with a breast cancer family history and an inconclusive *BRCA* genetic test have a higher risk of developing breast cancer (RR = 3.94, 95 % CI 3.09–5.02), but no increased ovarian cancer risk [27].

Monitoring of breast cancer for these women should include breast awareness from age 18 (**III**,**B**), clinical breast examination every 6 months since age 25 (**III**,**B**) and annual mammography from age 40 or 10 years before the youngest case of breast cancer in the family (**II**,**B**). Add an annual breast MRI from age 25 when the BC lifetime risk is over 20–25 % (**IIB**). The risk will be determined by predictive models as BRCAPRO, BOADICEA or Tyrer-Cuzyck [28] (Table 4).

**Table 4:** Surveillance in women from high-risk families without identified BRCA mutations

Breast self awareness starting at age 18 (**IIIB**)
Semiannual clinical breast exam starting at age 25 (**IIIB**)
Annual mammogram starting at age 40–70, or 10 years before the youngest age of BC in the family (**IIB**)
If lifetime risk >20–25 %, consider annual breast MRI starting at age 25 (**IIB**)
Ovarian early detection is not necessary in women with no family history of ovarian carcinoma (**IIA**)

Surveillance with ultrasound should not routinely be offered to women at moderate or high risk of BC, but it might be considered when MRI surveillance is not suitable or when results of mammography or MRI are difficult to interpret.

Gynaecological monitoring is not necessary in families with no family history of ovarian carcinoma. If family history of ovarian carcinoma exists, the medical management should be individualised.

Tamoxifen (pre- and post-menopausal women) and raloxifene (only postmenopausal women) are recommended for breast cancer chemoprevention for a maximum of 5 years among women at high risk according to the NICE guidelines (http://www.nice.org.uk/guidance/cg164), and are approved by the FDA for this purpose. Both drugs can be considered for breast cancer chemoprevention in women >35 years at high risk (**I**,**A**).

## Other hereditary breast cancer syndromes

Other breast cancer susceptibility genes have been identified as *TP53*, *PTEN* and *PALB2* as high penetrance, and *CHEK2*, *BRIP1*, *ATM*, *BARD1*, *NBN*, *RAD51C* with lower penetrance [2]. *CDH1* is considered high-penetrance gene for diffuse gastric cancer, but the BC risk has not been fully established.

*TP53* is involved in only 1 % of hereditary breast cancer cases. It is associated with a high lifetime risk of cancer, most diagnosed at young age as bone and soft tissue sarcoma, premenopausal breast cancer, acute leukaemia, colon cancer, adrenal cortex carcinoma, brain tumours and ovarian cancer.

Breast screening must begin at age 20 years with annual MRI and add annual mammogram at age 30. The option of risk-reducing mastectomy should be discussed in *TP53* mutation carriers (**II**,**A**).

Cowden syndrome (*PTEN*) is a rare hereditary cancer syndrome. The incidence is 1 in 200.000. The estimated cumulative lifetime risk for breast cancer is 67–85 % and patients may develop cancers of thyroid, endometrial, colorectal, renal and melanoma. Hamartomatous lesions are characteristic manifestations of this syndrome.

Breast screening consists of annual mammogram and breast MRI beginning at age 30. The option of risk-reducing mastectomy and hysterectomy should be discussed (**II**,**A**).

Germline mutations in *CDH1* are associated with Hereditary Diffuse Gastric Cancer Syndrome [29]. Women have a cumulative lifetime risk of lobular breast cancer of 23–68 %. *CDH1* germline mutations can also be found in the absence of DG cancer, especially in families with >3 lobular BC or bilateral lobular cancer before age 50.

Annual mammography and breast MRI from the age of 35 is recommended in *CDH1* mutation carriers (**II**,**A**).

The breast cancer risk associated with mutations in the lower-penetrance genes is currently imprecise. Clinical management is case-by-case depending on the family history [30]. The use of multigene panel testing will increase the diagnosis of such mutations, but more clinical research is needed to learn about their precise clinical meaning.

**Evidence levels** To assign a level of evidence and a grade of recommendation to the different statements of this guideline, it was decided to use the Infectious Diseases Society of America-US Public Health Service Grading System for Ranking Recommendations in Clinical Guidelines to determine the quality of evidence and strength of recommendation in each of the consensus recommendations [31].
